# Integrative treatment program for the treatment of children with autism spectrum disorder: A prospective observational case series

**DOI:** 10.3389/fneur.2022.1017005

**Published:** 2023-01-04

**Authors:** Boram Lee, Serin Park, Hyun Jeong Kwon, Gwi Seo Hwang, Moonju Kim

**Affiliations:** ^1^KM Science Research Division, Korea Institute of Oriental Medicine, Daejeon, South Korea; ^2^Floortime Center Korea, Seoul, South Korea; ^3^College of Korean Medicine, Gachon University, Seongnam, South Korea; ^4^I-Tomato Korean Medicine Clinic, Seoul, South Korea

**Keywords:** integrative medicine, autism spectrum disorder, herbal medicine, Floortime, sensory enrichment therapy, case series

## Abstract

**Background:**

In a situation where conventional treatments for autism spectrum disorder (ASD) are labor-intensive and there are concerns about the side effects of conventional medications, a 6-month integrative treatment program, including herbal medicine (HM), Floortime, and sensory enrichment therapy (SET) has been used on children with ASD in Korean medicine clinical settings.

**Methods:**

We observed the treatment responses of 18 children with ASD (66.7% male, mean age 3.9 ± 0.9 years) to the integrative treatment program as part of a prospective, single-center, observational case series. Individualized HMs were administered according to the patient's symptoms, and parents were instructed to perform Floortime and SET with their children at home for 2 h and 20 min a day, 5 days a week, respectively. The Childhood Autism Rating Scale (CARS) and Autism Behavior Checklist (ABC) were used to evaluate the core symptoms of ASD. A linear mixed model for repeated measures was used for analyzing the effect of the program over time, and logistic regression used to explore the predictors of treatment response.

**Results:**

The CARS and ABC scores were significantly improved from 34.58 ± 6.27 and 69.28 ± 15.73 at baseline to 28.56 ± 6.05 and 39.67 ± 20.36 after 6 months (*p* < 0.0001, respectively). No serious adverse events (AEs) were reported, and compliance with HM, Floortime, and SET was high at >90%.

**Conclusion:**

This 6-month integrative treatment program appears to be a potentially effective, safe, and feasible option for children with ASD. Low baseline CARS scores may be predictors of higher treatment response.

## Introduction

Autism spectrum disorder (ASD) is a neurodevelopmental disorder characterized by disturbances in social communication and interaction, as well as limited and repetitive behaviors, interests, and activities (1). The onset of ASD symptoms is usually before 2 years of age, and it was estimated that an average of 1 in 44 (2.3%) 8-year-old children had ASD in 2018 (2). Since this disorder occurs from the beginning of the life cycle and the illness period is long in many cases, the burden of indirect costs such as loss of income and productivity, as well as direct costs such as medical expenses, is large (3).

The conventional treatments for ASD include behavioral therapy and medication. However, because behavioral therapies involve the use of extensive and costly resources, their access may be limited (4). In addition, adverse events (AEs) such as drowsiness, somnolence, anxiety, hypersalivation, and elevation of prolactin levels have been reported with risperidone (5). Therefore, approximately 88% of the geographically diverse sample of children with ASD in the United States of America (USA) have used complementary and integrative medicine (CIM) (6), either to treat various symptoms, including hyperactivity and inattention (7), or because of concerns about the side effects of conventional treatments (8).

Herbal medicine (HM), one of the components of CIM, has been used for the treatment of ASD for many years in clinical settings, resulting in the improvement of ASD, as reported in various studies (9). In addition, the effectiveness of parents' direct involvement in the treatment of patients with ASD has been recognized through several studies (10, 11), such involvement has the advantage of building a positive emotional relationship between parents and patients in their home environment (12). Among direct parental involvement interventions, developmental, individual difference, relationship-based (DIR)/Floortime™ therapy (Floortime), and sensory enrichment therapy (SET) have also been used for the treatment of ASD in the clinical setting (13), and the effectiveness of individual treatments has been reported in various studies (14, 15). Floortime was developed by Greenspan, an American pediatrician, and is a developmental, multi-disciplinary, play-based framework that emphasizes building connections with others and underlines the meaningful, spontaneous use of language and communication (16). This therapy involves asking parents to “get down on the floor” and play with their child for a specified period, following the child's lead during play sessions and extending what the child does to elicit as many reciprocal interactions as possible. Sensory enrichment therapy is a form of therapy that uses two or more senses simultaneously, which can significantly reduce discomfort experienced by the autistic brain (17). Sensory enrichment therapy increased the size and weight of rats' brains, enriching more brain cells, more connections, and a stronger auxiliary system to support enhanced brain activity (18). Sensory enrichment therapy provides step-by-step training methods tailored to each patient's treatment goals by a qualified therapist, covering sensory processing, learning, memory, eating habits, sleeping habits, communication, sociality, fine motor skills, self-awareness, emotions and behaviors, attention, and anxiety. Based on an algorithm through periodic questionnaires and goals, a kit consisting of 3–4 worksheets for sensory development optimized for each patient is provided once a week. The kit has the advantage of convenient accessibility because it can be carried out with items used at home daily and without special tools. The SET kit activates a new brain area by simultaneously stimulating two or more senses (e.g., olfactory, and visual). After parents are educated about various sensations and movements by a professional therapist, they directly use the kit to treat their children at home (17).

In Korean medicine clinical settings, an integrative treatment program, including HM, Floortime, and SET, has been used for many years. However, to the best of our knowledge, there is no report on this integrative treatment program for ASD, which reflects real-world practice. Therefore, we report the results of our integrative treatment program in children with ASD through a prospective observational study, identify factors related to the treatment response, and provide evidence for a more rigorous study design.

## Methods

### Study design

This is a prospective, single-center, pragmatic observational case series. This study was approved by the Institutional Review Board of Gachon University (1044396-201812-HR-223-01), written informed consent was obtained from all patients before enrolling in this study, and their clinical records and information were anonymized and de-identified before analysis, by assigning an individual identification code to each patient.

### Patients and eligibility

Eligible patients were recruited in the outpatient setting of the I-Tomato Korean Medicine Clinic (Seoul, Republic of Korea) from March 2019 to July 2021. The inclusion criteria were (1) children aged 2–5 years, and (2) children diagnosed with ASD through the Autism Diagnostic Observation Schedule, Second Edition (ADOS-2), and the Diagnostic and Statistical Manual of Mental Disorders, Fifth Edition (DSM-5). The exclusion criteria were as follows: (1) patients with neurological abnormalities such as cerebral palsy and Down syndrome, (2) patients diagnosed with other psychiatric diseases such as attention deficit hyperactivity disorder and schizophrenia, (3) those diagnosed with serious chronic disease, malignant tumors, and tuberculosis, (4) those who stopped taking risperidone or aripiprazole within 3 months of study initiation, and (5) patients who were judged to be inappropriate for participation in the research by the investigators (e.g., if no data were available for analysis because the evaluation was not performed on time).

### Integrative treatment program

This 6-month integrative treatment program consisted of HM, Floortime, and SET. Individualized HM treatment was conducted based on the symptoms because this study was a pragmatic observational study with the goal of collecting information on the integrative treatment used in real-world clinical settings. Based on previous research and clinical experience, Shihogyeji-tang was used as the basis when sensory processing disorders were evident (19), Galgeun-tang when developmental dyspraxia was evident (20, 21), and Baekho-tang or Daeseunggi-tang when concentration disorders were evident (22, 23) (Table 1). Herbal medicine was prescribed for a total of 6 months, and the main prescriptions and dosages of individual herbs were changed, each month, according to patients' symptoms, and based on the judgment of a clinician with more than 20 years of clinical experience. Herbal medicine was administered in the form of a decoction, three times a day for a total volume of 40 ml per day. The type and dose of HMs used for individual patients were collected through case report forms (CRFs). Floortime was conducted for 1 h once a week with a certified professional therapist in consideration of the patient's individual developmental competency and profile. Parents were instructed to conduct Floortime with their children at home for more than 2 h a day, 5 days a week. Before the first session, all parents completed 12 h of an online training workshop from Floortime Center Korea (Seoul, Republic of Korea) to learn about Floortime. For SET, a certified professional therapist educated the parents once a week about patient-individualized counseling and play methods, including multi-sensory core training methods, such as smell, sight, touch, and taste. The parents were instructed to proceed with SET for more than 20 min a day (and on more than 5 days a week) at home with their children. The use of concomitant drugs was permitted if the investigator judged it necessary for patient treatment, and all drugs used during the observation period were recorded in the CRF.

**Table 1:** Details of herbal medicine used.

**Herbal medicine**	**Composition and doses (per day)**	**No. of patients (%)**
Shihogyeji-tang	Bupleuri Radix 6 g, Scutellariae Radix 3 g, Pinelliae Tuber 3 g, Ginseng Radix 3 g, Cinnamomi Ramulus 4 g, Paeoniae Radix 6 g, Zingiberis Rhizoma Recens 4 g, Zizyphi Fructus 4 g, Glycyrrhizae Radix et Rhizoma 2 g	17 (94.4)
Galgeun-tang	Cinnamomi Ramulus 4 g, Paeoniae Radix 4 g, Zingiberis Rhizoma Recens 4 g, Zizyphi Fructus 4 g, Glycyrrhizae Radix et Rhizoma 2 g, Puerariae Radix 5 g, Ephedrae Herba 1 g	17 (94.4)
Baekho-tang	Anemarrhenae Rhizoma 5 g, Oryzae Semen 8 g, Gypsum Fibrosum 15 g, Glycyrrhizae Radix et Rhizoma 2 g	6 (33.3)
Daeseunggi-tang	Rhei Radix et Rhizoma 2 g, Ponciri Fructus Immaturus 3 g, Natrii Sulfas 3 g, Magnoliae Cortex 5 g	2 (11.1)

### Outcome measurement

The Korean versions of the Childhood Autism Rating Scale (CARS) and Autism Behavior Checklist (ABC) were used to assess the severity of the ASD symptoms, while the Social Maturity Scale (SMS) was used to assess social maturity, with three measurements at baseline, 3 and 6 months after treatment. The CARS is a validated questionnaire consisting of 15 items related to the core symptoms of ASD. It is a tool with high reliability, validity, and inter-rater consistency, with a total cutoff score of 30 that distinguishes ASD from other developmental disorders (24, 25). The ABC is a 57-item tool designed to distinguish children with ASD from normal children and children with intellectual disabilities, hearing impairments, emotional disabilities. A total score of 67 or higher is classified as ASD (26). The SMS is a questionnaire consisting of 117 questions in 6 behavioral areas, including self-help, movement, work, communication, self-management, and socialization, and the social quotient is calculated based on the results (27). In addition, the Sequenced Language Scale for Infants (SELSI), a language evaluation tool, and ADOS-2, a tool for diagnosing ASD, were measured twice at baseline and 6 months after treatment by clinical psychologists. Symptom improvement is indicated by a low score in the case of CARS, ABC, and ADOS-2 but a high score for the SMS social quotient and SELSI. The ADOS-2 test results are classified into three categories according to the cut-off score: (1) autism, (2) ASD, or (3) non-ASD. According to these ADOS-2 classifications, responders are patients diagnosed with: (i) ASD or non-ASD after 6 months of treatment, following a diagnosis of baseline autism; or (ii) non-ASD after 6 months of treatment, following a diagnosis of baseline ASD. Assessment of the questionnaires used in this study was performed by the same qualified clinical psychologist or speech-language therapist, independent of the integrative treatment program.

For safety evaluation, adverse reactions were monitored based on physical examination and patient reports during the observation period, and the severity and causal relationship with treatment was recorded in detail in the CRF. Based on previous studies (28, 29), we considered the following as possible AEs related to HM: digestive system disorders (including indigestion, nausea, and diarrhea), nervous system disorders (including headache, fatigue, and dizziness), mental and behavior disorders (including loss of appetite, increased appetite, hypomania, and sedation), skin and subcutaneous tissue disorders (including rash and burning sensation of the skin), circulatory system disorders (including mild hypoglycemia, mild paroxysmal palpitation, and increased palpitation), respiratory system disorders (including upper respiratory tract infection), genitourinary system disorders (including uterine bleeding and dysuria), nutritional and metabolism disorders (including weight loss and dysgeusia), and others. Some AEs were considered by the investigator to have a causal relationship with the HM, based on the temporal relationship with treatment. Adverse events were classified as follows: mild (does not interfere with the patient's normal daily life and causes minimal discomfort), moderate (causes discomfort that significantly interferes with the patient's normal daily life), and severe (the patient's normal daily activities are impossible). Program adherence was calculated by collecting information from parents on performance rates for individual treatments, including HM, Floortime, and SET, during the observation period.

### Statistical analysis

A linear mixed-effects model for repeated measures was used for assessing effect changes at baseline, 3 months, and 6 months after treatment; a Dunnett *post-hoc* test was conducted for multi-group comparison. The effect changes at baseline and after 6 months were statistically analyzed using the two-sided paired *t*-test or Wilcoxon signed-rank test after a data normality test using a Shapiro-Wilk test. Categorical variables were expressed as frequency (percentage), and continuous variables were expressed as mean (standard deviation) or median (interquartile range) according to the normality of data. In addition, baseline characteristics predicting treatment responders were derived through logistic regression analysis, and univariable and multivariable odds ratios (ORs) and 95% confidence interval (CIs) were calculated. A *p*-value < 0.05 was considered statistically significant. All statistical analysis was performed using R statistical software version 4.1.2 (for windows).

## Results

### Baseline characteristics

A total of 19 patients were screened for eligibility during the observation period, and a total of 18 patients were enrolled for the study, excluding one patient who was non-ASD on the ADOS-2 test. All enrolled patients participated in and completed the integrative treatment program for 6 months, and the treatment effect was evaluated every 3 months. Twelve of 18 patients (66.7%) were male, and the mean age and body mass index (BMI) of all patients were 3.9 ± 0.9 years and 22.0 ± 4.0, respectively. According to the ADOS-2 classification, 10 patients (55.6%) were identified to have autism, and 8 patients (44.4%) had ASD (Table 2). There was no patient with any clinically significant medical history or surgical history other than ASD. No patients received concomitant conventional medications, including risperidone, during the observation period.

**Table 2:** Baseline demographic and clinical characteristics of patients.

**Characteristics**	**Total (*n* = 18)^†^**
Sex (Male/Female)	12 (66.7%)/6 (33.3%)
Age (year)	3.9 ± 0.9
Height (cm)	101.1 ± 8.6
Weight (kg)	17.1 ± 2.6
BMI (kg/cm^2^)	22.0 ± 4.0
ADOS-2 (Autism/ASD)	10 (55.6%)/8 (44.4%)
ADOS-2	17.9 ± 8.1
CARS	34.6 ± 6.3

ADOS-2, autism diagnostic observation schedule, second edition; ASD, autism spectrum disorder; BMI, body mass index; CARS, childhood autism rating scale.

† Number (%) or mean ± standard deviation.

### Integrative treatment program and compliance rates

Herbal medicine was prescribed by a Korean medicine doctor and administered by parents three times a day for 6 months, according to the individual symptoms. Shihogyeji-tang- and Galgeun-tang-based prescriptions were used in 17 patients (each 94.4%), Baekho-tang-based prescription was used in 6 patients (33.3%), and Daeseunggi-tang based prescription was used in 2 patients (11.1%) (Table 1). The average compliance rate for HMs was 98.0% ± 4.4%. The parents of the patients were educated to perform Floortime and SET at least 5 times a week, for at least 2 h and 20 min a day, respectively. Based on this, the average performance rates of Floortime and SET were 94.9% ± 10.0% and 90.8% ± 23.3%, respectively.

### Effect assessment

**Figure 1:**
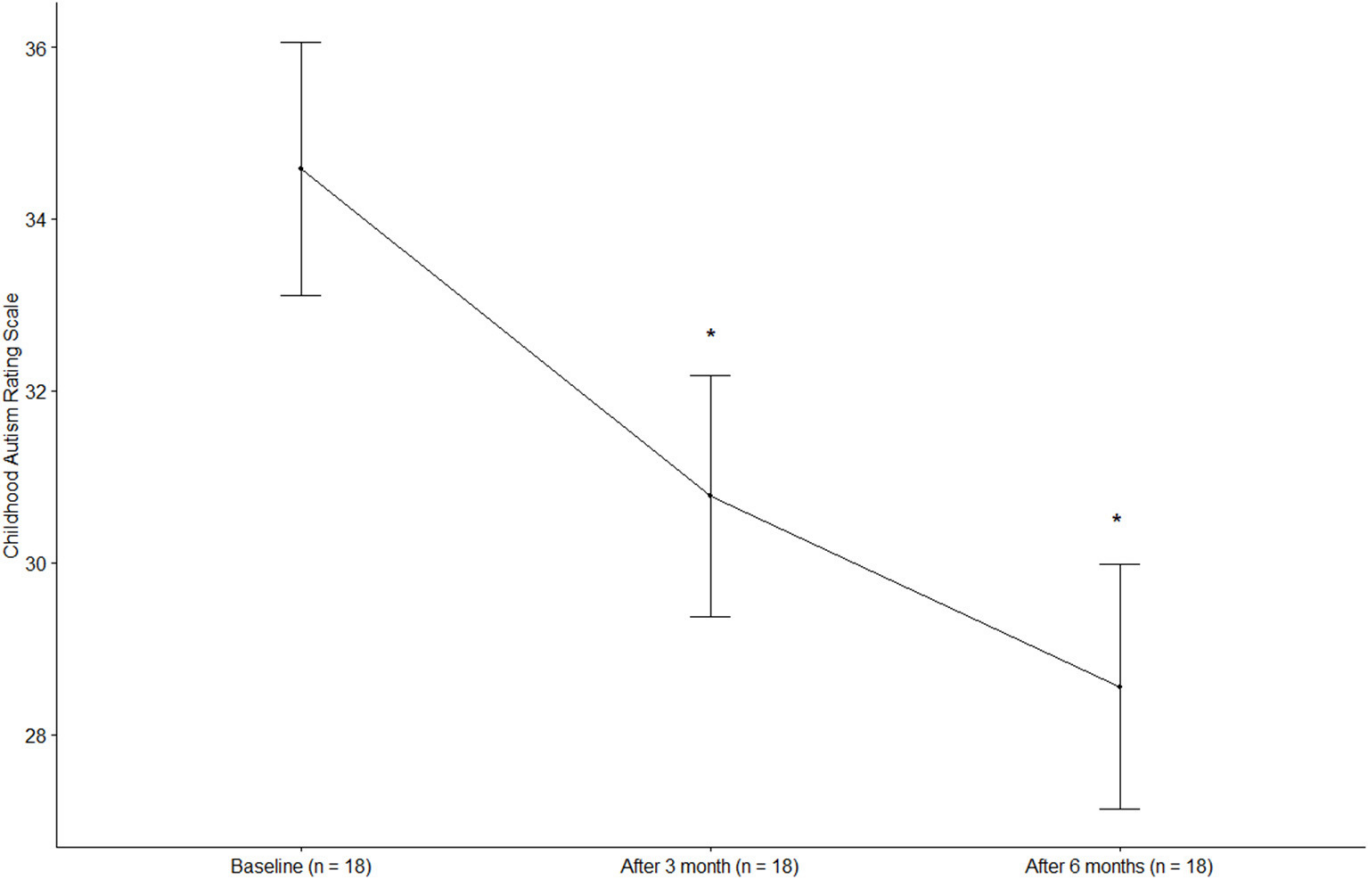
Changes in the childhood autism rating scale. *p < 0.0001, compared with baseline (Dunnett *post-hoc* test).

The CARS score was 34.58 ± 6.27 (mean ± standard deviation) at baseline and decreased significantly to 30.78 ± 5.95 after 3 months and 28.56 ± 6.05 after 6 months (*p* < 0.0001) (Figure 1). The total ABC score was 69.28 ± 15.73 at baseline and decreased significantly to 46.44 ± 19.14 after 3 months and 39.67 ± 20.36 after 6 months (*p* < 0.0001). The ABC subscales, including sensory, relating, body and object use, language, social, and self-help skills, all improved significantly at 3 and 6 months of treatment compared to the baseline (*p* < 0.05, all). The social quotient calculated by SMS after 6 months of treatment was higher than that at baseline (*p* = 0.0627). In the case of language ability measured by SELSI, both receptive and expressive language scores increased after treatment compared to the baseline, with a significant increase observed only in expressive language (*p* = 0.0069). The ADOS-2 score was significantly decreased after 6 months of treatment (12.39 ± 6.96) compared to the baseline (17.89 ± 8.11) (*p* = 0.002) (Table 3). After 6 months of treatment, the classification of ADOS-2 was as follows: autism in 3 patients (16.7%), ASD in 11 patients (61.1%), and non-ASD in 4 patients (22.2%). There were 9 (50%) treatment responders, as defined by the ADOS-2 classification change. In multivariate logistic regression analysis of the characteristics predicting treatment response, the lower the baseline CARS score, the higher was the treatment response (OR 0.65, 95% CI 0.30–0.98). Other characteristics such as age, sex, height, weight, BMI, and ADOS-2 score did not affect the treatment response (Table 4).

**Table 3:** Changes in outcome measures.

**Outcomes**	**Baseline** **(*n* = 18)**	**After 3 months**	**After 6 months**	***p*** **-values**	
		**(*n* = 18)**	**(*n* = 18)**	**Baseline vs. 3 months**	**Baseline vs. 6 months**
CARS^†^	34.58 ± 6.27	30.78 ± 5.95	28.56 ± 6.05	< 0.0001	< 0.0001
ABC total^†^	69.28 ± 15.73	46.44 ± 19.14	39.67 ± 20.36	< 0.0001	< 0.0001
ABC sensory^†^	10.28 ± 3.95	5.00 ± 4.91	3.44 ± 3.57	< 0.0001	< 0.0001
ABC relating^†^	17.72 ± 6.69	10.22 ± 5.44	9.00 ± 6.41	< 0.0001	< 0.0001
ABC body and object use^†^	11.06 ± 5.10	8.06 ± 5.96	6.61 ± 5.91	0.0369	0.0011
ABC language^†^	18.61 ± 6.33	15.22 ± 6.69	12.17 ± 6.83	0.0015	< 0.0001
ABC social and self-help^†^	11.61 ± 5.82	7.94 ± 3.84	8.44 ± 4.59	< 0.0001	0.0003
SMS social quotient^†^	70.42 ± 15.79	73.82 ± 18.78	76.65 ± 26.26	0.4038	0.0627
SELSI receptive language score^‡^	26.00 (32.00)	–	29.50 (31.00)	–	0.0779
SELSI expressive language score^†^	27.67 ± 17.09	–	32.78 ± 16.10	–	0.0069
ADOS-2^†^	17.89 ± 8.11	–	12.39 ± 6.96	–	0.002

ABC, autism behavior checklist; ADOS-2, autism diagnostic observation schedule, second edition; CARS, childhood autism rating scale; SELSI, sequenced language scale for infants; SMS, social maturity scale.

† Mean ± standard deviation,

‡ median (interquartile).

**Table 4:** Logistic regression analysis: characteristics predicting treatment response.

**Characteristics**		**Non-responder (*n* = 9)^†^**	**Responder (*n* = 9)^†^**	**Univariable**	**Multivariable**
				**OR (95% CI)**	**OR (95% CI)**
Age (year)		3.9 ± 0.9	4.0 ± 0.9	1.14 (0.37–3.71)	3.37 (0.45–76.74)
Sex	Female	2 (33.3%)	4 (66.7%)	–	–
	Male	7 (58.3%)	5 (41.7%)	0.36 (0.04–2.61)	1.34 (0.06–50.86)
Height (cm)		102.6 ± 7.4	99.6 ± 9.8	0.96 (0.84–1.07)	2.02 (0.52–12.34)
Weight (kg)		17.6 ± 2.7	16.7 ± 2.6	0.88 (0.58–1.27)	1.83 (0.71–7.92)
BMI (kg/cm^2^)		21.2 ± 3.1	22.8 ± 4.8	1.12 (0.88–1.49)	8.44 (0.49–727.80)
ADOS-2		17.1 ± 9.8	18.7 ± 6.5	1.03 (0.91–1.17)	1.29 (0.97–2.10)
CARS		36.0 ± 5.9	33.2 ± 6.7	0.92 (0.76–1.08)	0.65 (0.30–0.98)

ADOS-2, autism diagnostic observation schedule, second edition; BMI, body mass index; CARS, childhood autism rating scale; CI, confidence interval; OR, odds ratio.

† Number (%) or mean ± standard deviation.

### Safety assessment

During the observation period, a total of three AEs occurred in two patients: loose stool, constipation, and difficulty falling asleep. All AEs suspected of having a causal relationship with the HMs were mild because the AEs did not interfere with the patients' normal daily lives and caused minimal discomfort, and the AEs improved spontaneously without any treatment; therefore, these AEs were judged to have no effect on the study results.

## Discussion

Autism spectrum disorder is a disease with increasing prevalence and a large social and economic burden. Behavioral therapy—the conventional treatment—is labor-intensive and costly, while there are concerns about the side effects of conventional medications. Therefore, the integrative treatment program, including HM, Floortime, and SET, has been used for many years in Korean medicine clinical practice. Therefore, we aimed to provide the first report of the effectiveness, safety, and feasibility of the program in this prospective, observational study, and confirmed the predictive variables for the treatment response. Our study showed that a 6-month integrative treatment program resulted in a significant improvement of ASD symptoms and language ability, with a high program compliance rate and no serious AEs.

In particular, CARS and ABC scores dropped from respective averages of 34.58 and 69.28 at baseline to 28.56 and 39.67 after 6 months of treatment; both 6-month scores were below the ASD cut-off scores of 30 and 67, respectively (25, 26). Considering that the minimal clinically important difference for CARS score after an intervention has been defined as 4.5 points (30), an improvement of 6.02 points in CARS, which evaluates the core symptoms of ASD, after the integrative treatment program means a significant improvement. As a result of regression analysis of the patients' characteristics predicting the treatment response as defined by the ADOS-2 classification change, only the baseline CARS score was found to affect the treatment response. It was found that the lower the baseline CARS score, the higher was the treatment response. This means that if ASD is detected earlier and the integrative treatment program is implemented earlier, the treatment effect will be higher. This result is consistent with the current guidelines that emphasize the importance of early detection of ASD and intensive treatment (31).

In this study, different basic HM prescriptions were used according to the main symptoms of each patient. Shihogyeji-tang, Galgeun-tang, and Baekho-tang or Daeseunggi-tang were used as the basic prescriptions for sensory processing disorder, developmental dyspraxia, and concentration disorder, respectively. In a previous study, the use of modified Shihogyeji-tang in 21 autistic patients resulted in a significant improvement in the sensory integration ability compared to before treatment (19). In addition, there have been studies showing the effect of Galgeun-tang on dystonia (20, 21) and of modified Daeseunggi-tang and Baekho-tang on attention deficit hyperactivity disorder (22, 23). It has been reported that functional emotional development and symptom severity were significantly improved when performing home-based Floortime for preschool children with ASD (32). Furthermore, several clinical trials have reported significant improvements in ASD severity, cognitive ability, sensory response, and language in the group receiving SET compared to children who did not receive SET (15, 33). Although the effects of individual treatments for the treatment of ASD have been determined by various researchers, to the best of our knowledge, our study is the first to report the effectiveness of this integrative treatment program. In addition, according to our study, program compliance was very high—>90% for all components—showing that it was sufficiently feasible in the clinical field.

Although research on the treatment mechanism of this integrative treatment program has not been conducted yet, the mechanisms of the individual treatments have been explored in several studies. The individual herbs of the HM prescriptions used in this program are used frequently for the treatment of ASD, ameliorate abnormal behavior in animal models of ASD, and have sedative and anticonvulsant effects (34–36). It has been reported that a shift occurs from the ventral to the dorsal systems in the medial prefrontal cortex in the brain of children with ASD after Floortime, suggesting that the children are gaining more cognitive control over their emotional system using such executive functions as response inhibition and self-monitoring (37). In addition, several animal studies have reported that SET changes the neuroplasticity of the brain. Sensory enrichment therapy resulted in rat cortical growth and an improvement in learning and motor memory (38). Furthermore, SET reduced neurological deficits, increased nerve dendrites, nerve branch density, nerve synapses, and surrounding blood vessels, as well as brain weight and size (39–42). Although the pathogenesis of ASD has not yet been clearly elucidated, recent studies have reported a link between abnormalities in brain regions and the core symptoms of ASD (43, 44). Therefore, it can be estimated that the therapeutic effect of this integrative treatment program was derived from the combination of these individual treatment mechanisms, warranting further research on the overall mechanism of the integrative treatment program.

This study has several limitations. First, since this study was a prospective observational study and not a controlled intervention study, it is not possible to draw clear conclusions about the reported effects. Although we confirmed that no other conventional medication was used during the observation period in the included patients, there may be other potential effects that could have affected the study outcome. Second, the safety assessment of the program relied solely on patient reports and physical examination. Considering that HMs were included in the treatment program, it would be helpful to report the safety of the program using laboratory tests in future studies. Finally, although adherence to individual treatment programs was >90%, on average, this was confirmed from parental reports. Because parents' adherence to the programs can have a great impact on study results, future research should aim to increase and systematically check parents' adherence.

Nevertheless, because conventional treatment for ASD is labor-intensive and expensive, and there are concerns about side effects of conventional medication, such an integrative treatment program would be highly beneficial. However, as there have been no studies on the effect of this integrative treatment program, we report the first case series on this integrative treatment program that is frequently used in Korean medicine clinical settings. We evaluated the therapeutic effect of HMs, Floortime, and SET on ASD using validated questionnaires and diagnostic tools, reduced related biases, and increased the reliability of the study results through a prospective observational study design. In particular, this is a treatment program in which the role of the parents is important, and as it can be conducted non-face-to-face in the “untact era,” it is free from time and space constraints. In addition, there is a possibility that the therapeutic effect may be increased by increasing the role of the parents in performing Floortime and SET. Given the promising results of our study, further rigorous, well-designed controlled trials with a larger sample size should be conducted to confirm the clinical effectiveness of such integrative treatment programs.

## Conclusion

A 6-month integrative treatment program, including HM, Floortime, and SET, could help improve the core symptoms of ASD with a high compliance rate and no serious AEs. Future well-designed, prospective randomized controlled trials with a larger sample size should be conducted to assess the effectiveness of the program.

## Data availability statement

The raw data supporting the conclusions of this article will be made available by the authors, without undue reservation.

## Ethics statement

The studies involving human participants were reviewed and approved by the Institutional Review Board of Gachon University (1044396-201812-HR-223-01). Written informed consent to participate in this study was provided by the participants' legal guardian/next of kin.

## Author contributions

MK: conception and design of the study. SP, HK, and MK: acquisition of data. BL: analysis of data and drafting the manuscript or figures. SP, HK, GH, and MK: critical review and edits. All authors contributed to the article and approved the submitted version.

## References

[1] American Psychiatric Association. Diagnostic and Statistical Manual of Mental Disorders. Washington, DC: American Psychiatric Publishing. 10.1176/appi.books.9780890425596

[2] Maenner MJ Shaw KA Bakian AV Bilder DA Durkin MS Esler A. Prevalence and characteristics of autism spectrum disorder among children aged 8 years - autism and developmental disabilities monitoring network, 11 sites, United States, 2018. MMWR Surveill Summ. (2021) 70:1–16. 10.15585/mmwr.ss7011a1 34855725 PMC8639024

[3] Baxter AJ Brugha TS Erskine HE Scheurer RW Vos T Scott JG. The epidemiology and global burden of autism spectrum disorders. Psychol Med. (2015) 45:601–13. 10.1017/S003329171400172X 25108395

[4] Masi A Demayo MM Glozier N Guastella AJ. An overview of autism spectrum disorder, heterogeneity and treatment options. Neurosci Bull. (2017) 33:183–93. 10.1007/s12264-017-0100-y 28213805 PMC5360849

[5] Maneeton N Maneeton B Putthisri S Woottiluk P Narkpongphun A Srisurapanont M. Risperidone for children and adolescents with autism spectrum disorder: a systematic review. Neuropsychiatr Dis Treat. (2018) 14:1811–20. 10.2147/NDT.S151802 30022830 PMC6045903

[6] Owen-Smith AA Bent S Lynch FL Coleman KJ Yau VM Pearson KA. Prevalence and predictors of complementary and alternative medicine use in a large insured sample of children with autism spectrum disorders. Res Autism Spectr Disord. (2015) 17:40–51. 10.1016/j.rasd.2015.05.002 26366192 PMC4562462

[7] Klein N Kemper KJ. Integrative approaches to caring for children with autism. Curr Probl Pediatr Adolesc Health Care. (2016) 46:195–201. 10.1016/j.cppeds.2015.12.004 26776326

[8] Hanson E Kalish LA Bunce E Curtis C McDaniel S Ware J. Use of complementary and alternative medicine among children diagnosed with autism spectrum disorder. J Autism Dev Disord. (2007) 37:628–36. 10.1007/s10803-006-0192-0 16977497

[9] Bang M Lee SH Cho SH Yu SA Kim K Lu HY. Herbal medicine treatment for children with autism spectrum disorder: a systematic review. Evid Based Complement Alternat Med. (2017) 2017:8614680. 10.1155/2017/8614680 28592982 PMC5448044

[10] Kasari C Gulsrud AC Wong C Kwon S Locke J. Randomized controlled caregiver mediated joint engagement intervention for toddlers with autism. J Autism Dev Disord. (2010) 40:1045–56. 10.1007/s10803-010-0955-5 20145986 PMC2922697

[11] Schertz HH Horn K Lee M Mitchell S. Supporting parents to help toddlers with autism risk make social connections. Young Except Child. (2017) 20:16–29. 10.1177/1096250615576808

[12] Green J Wan MW Guiraud J Holsgrove S McNally J Slonims V. Intervention for infants at risk of developing autism: a case series. J Autism Dev Disord. (2013) 43:2502–14. 10.1007/s10803-013-1797-8 23532347

[13] Sandbank M Bottema-Beutel K Crowley S Cassidy M Dunham K Feldman JI. Project AIM: autism intervention meta-analysis for studies of young children. Psychol Bull. (2020) 146:1–29. 10.1037/bul0000215 31763860 PMC8783568

[14] Casenhiser DM Shanker SG Stieben J. Learning through interaction in children with autism: preliminary data from asocial-communication-based intervention. Autism. (2013) 17:220–41. 10.1177/1362361311422052 21949005

[15] Woo CC Leon M. Environmental enrichment as an effective treatment for autism: a randomized controlled trial. Behav Neurosci. (2013) 127:487–97. 10.1037/a0033010 23688137

[16] Greenspan SI Wieder S. A functional developmental approach to autism spectrum disorders. J Assoc Pers Severe Handicaps. (1999) 24:147–61. 10.2511/rpsd.24.3.147

[17] Aronoff E Hillyer R Leon M. Environmental enrichment therapy for autism: outcomes with increased access. Neural Plast. (2016) 2016:2734915. 10.1155/2016/2734915 27721995 PMC5046013

[18] Krech D Rosenzweig MR Bennett EL. Effects of environmental complexity and training on brain chemistry. J Comp Physiol Psychol. (1960) 53:509. 10.1037/h0045402 13754181

[19] Li Y. Treatment of 21 cases of autism with the main formula of Chaihu Jiaguizhi Longgu Muli decoction. Chin Med Mod Dist Educ China. (2011) 9:57–8. 10.3969/j.issn.1672-2779.2011.22.039

[20] Wang G. Epidemic myodystonia syndrome treated by integrated traditional and western medicine: report of 88 cases. J Tradit Chin Med. (1994) 35:544–5.

[21] Zhu E. One case of Meige syndrome treated by Gegen decoction. Inner Mong J Tradit Chin Med. (2018) 37:27–8. 10.16040/j.cnki.cn15-1101.2018.02.021

[22] Xiang H Du Y. One case of infantile ADHD treated by modified Xiaochengqi decoction. Guangxi J Tradit Chin Med. (2008) 31:45.

[23] Ding L Li CJ Jin D. Experience on the treatment for infantile attention-deficit hyperactivity disorder by Chinese medicine. Chin Pediatr Integr Tradit West Med. (2015) 7:502–4. 10.3969/j.issn.1674-3865.2015.05.034

[24] Schopler E Reichler RJ Devellis RF Daly K. Toward objective classification of childhood autism: Childhood Autism Rating Scale (CARS). J Autism Dev Disord. (1980) 10:91–103. 10.1007/BF02408436 6927682

[25] Shin MS Kim Y-H. Standardization study for the Korean version of the Childhood Autism Rating Scale: reliability, validity and cut-off score. Kor J Clin Psychol. (1998) 17:1–15.

[26] Krug DA Arick J Almond P. Behavior checklist for identifying severely handicapped individuals with high levels of autistic behavior. J Child Psychol Psychiatry. (1980) 21:221–9. 10.1111/j.1469-7610.1980.tb01797.x 7430288

[27] Kim SG Kim OG. Social Maturity Scale Protocol. Seoul: Jungang Aptitude Laboratory (1995).

[28] Lee JY Jun SA Hong SS Ahn YC Lee DS Son CG. Systematic review of adverse effects from herbal drugs reported in randomized controlled trials. Phytother Res. (2016) 30:1412–9. 10.1002/ptr.5647 27196988

[29] Jang S Kim KH Sun SH Go HY Lee EK Jang BH. Characteristics of herbal medicine users and adverse events experienced in south korea: a survey study. Evid Based Complement Alternat Med. (2017) 2017:4089019. 10.1155/2017/4089019 28491107 PMC5402245

[30] Jurek L Baltazar M Gulati S Novakovic N Núñez M Oakley J. Response (minimum clinically relevant change) in ASD symptoms after an intervention according to CARS-2: consensus from an expert elicitation procedure. Eur Child Adolesc Psychiatry. (2022) 31:1–10. 10.1007/s00787-021-01772-z 33825947 PMC8024930

[31] Sanchack KE Thomas CA. Autism spectrum disorder: primary care principles. Am Fam Physician. (2016) 94:972–9. 28075089

[32] Pajareya K Nopmaneejumruslers K. A pilot randomized controlled trial of DIR/Floortime™ parent training intervention for pre-school children with autistic spectrum disorders. Autism. (2011) 15:563–77. 10.1177/1362361310386502 21690083

[33] Woo CC Donnelly JH Steinberg-Epstein R Leon M. Environmental enrichment as a therapy for autism: a clinical trial replication and extension. Behav Neurosci. (2015) 129:412–22. 10.1037/bne0000068 26052790 PMC4682896

[34] Gonzales EL Jang JH Mabunga DF Kim JW Ko MJ Cho KS. Supplementation of Korean Red Ginseng improves behavior deviations in animal models of autism. Food Nutr Res. (2016) 60:29245. 10.3402/fnr.v60.29245 26837496 PMC4737717

[35] Lee J Son K Hwang G Kim M. Effect and safety of shihogyejitang for drug resistant childhood epilepsy. Evid Based Complement Alternat Med. (2016) 2016:3410213. 10.1155/2016/3410213 27047568 PMC4800883

[36] Jiang H Yang L Hou A Zhang J Wang S Man W. Botany, traditional uses, phytochemistry, analytical methods, processing, pharmacology and pharmacokinetics of Bupleuri Radix: a systematic review. Biomed Pharmacother. (2020) 131:110679. 10.1016/j.biopha.2020.110679 32858498

[37] Stieben J Lewis MD Granic I Zelazo PD Segalowitz S Pepler D. Neurophysiological mechanisms of emotion regulation for subtypes of externalizing children. Dev Psychopathol. (2007) 19:455–80. 10.1017/S0954579407070228 17459179

[38] Van Praag H Kempermann G Gage FH. Neural consequences of environmental enrichment. Nat Rev Neurosci. (2000) 1:191–8. 10.1038/35044558 11257907

[39] Briones TL Therrien B Metzger B. Effects of environment on enhancing functional plasticity following cerebral ischemia. Biol Res Nurs. (2000) 1:299–309. 10.1177/109980040000100406 11232208

[40] Ben-Sasson A Hen L Fluss R Cermak SA Engel-Yeger B Gal E. A meta-analysis of sensory modulation symptoms in individuals with autism spectrum disorders. J Autism Dev Disord. (2009) 39:1–11. 10.1007/s10803-008-0593-3 18512135

[41] Baroncelli L Braschi C Spolidoro M Begenisic T Sale A Maffei L. Nurturing brain plasticity: impact of environmental enrichment. Cell Death Differ. (2010) 17:1092–103. 10.1038/cdd.2009.193 20019745

[42] Mering S Jolkkonen J. Proper housing conditions in experimental stroke studies-special emphasis on environmental enrichment. Front Neurosci. (2015) 9:106. 10.3389/fnins.2015.00106 25870536 PMC4378295

[43] Amaral DG Schumann CM Nordahl CW. Neuroanatomy of autism. Trends Neurosci. (2008) 31:137–45. 10.1016/j.tins.2007.12.005 18258309

[44] Hazlett HC Gu H Munsell BC Kim SH Styner M Wolff JJ. Early brain development in infants at high risk for autism spectrum disorder. Nature. (2017) 542:348–51. 10.1038/nature21369 28202961 PMC5336143

